# RNA-seq analysis reveals significant effects of EGFR signalling on the secretome of mesenchymal stem cells

**DOI:** 10.18632/oncotarget.2420

**Published:** 2014-10-29

**Authors:** Antonella De Luca, Cristin Roma, Marianna Gallo, Francesca Fenizia, Francesca Bergantino, Daniela Frezzetti, Susan Costantini, Nicola Normanno

**Affiliations:** ^1^ Cell Biology and Biotherapy Unit, Istituto Nazionale Tumori “Fondazione G. Pascale”-IRCCS, Naples, Italy; ^2^ Centro di Ricerche Oncologiche di Mercogliano (CROM)-Istituto Nazionale Tumori “Fondazione G. Pascale”-IRCCS, Mercogliano (AV), Italy

**Keywords:** EGFR, mesenchymal stem cells, tumor microenvironment, RNA-seq

## Abstract

Bone marrow-derived mesenchymal stem cells (MSCs) contribute to breast cancer progression by releasing soluble factors that sustain tumor progression. MSCs express functional epidermal growth factor receptor (EGFR) and breast cancer cells secrete EGFR-ligands including transforming growth factor-α (TGFα). Using RNA-sequencing, we analysed the whole transcriptome of MSCs stimulated with TGFα. We identified 1,640 highly differentially regulated genes: 967 genes up-regulated with Fold Induction (FI)≥1.50 and 673 genes down-regulated with FI≤0.50. When highly regulated genes were categorized according to GO molecular function classification and KEGG pathways analysis, a large number of genes coding for potentially secreted proteins or surface receptors resulted enriched following TGFα treatment, including *VEGFA, IL6, EREG, HB-EGF, LIF, NGF, NRG1, CCL19, CCL2, CCL25* and *CXCL3*. Secretion of corresponding proteins was confirmed for selected factors. Finally, we identified 4,377 and 4,262 alternatively spliced genes in untreated and TGFα-treated MSCs, respectively. Among these, an unannotated splice variant of *VEGFA* coding for a secreted VEGF protein of 172 aminoacids (VEGFA_172_), was found only in MSCs stimulated with TGFα. These findings suggest that EGFR activation in MSCs leads to a significant change in the expression of a wide array of genes coding for secreted proteins that can significantly enhance tumor progression.

## INTRODUCTION

Several studies demonstrated that bone marrow-derived mesenchymal stem cells (MSCs) contribute to breast cancer progression [1]. In particular, MSCs secrete a number of growth factors, chemokines and cytokines that sustain breast cancer cell proliferation, survival and invasion and that are able to modulate functions of the tumor microenvironment essential for tumor growth including angiogenesis. In this respect, the chemokine (C-C motif) ligand 5 (CCL5/RANTES), the monocyte chemotactic protein-1 (MCP-1/CCL2), interleukin 17B (IL17B) and the chemokine (C-X-C motif) ligand 10 (CXCL10) are known to be involved in the cross-talk between MSCs and breast cancer cells and to favour the development of metastases [2–5]. Moreover, we recently demonstrated that different factors produced by MSCs, such as vascular endothelial growth factor A (VEGF) and IL6, may cooperate in promoting breast cancer cell migration [6].

Despite a number of MSCs-secreted factors have been demonstrated to be involved in the cross-talk between MSCs and breast cancer cells, little information is available on the factors released by breast cancer cells that might regulate the secretome of MSCs.

Epidermal growth factor (EGF)-like growth factors bind and activate the ErbB family of tyrosine kinase receptors that comprises four distinct members, EGFR, ErbB-2, ErbB-3 and ErbB-4 [7, 8]. Following ligand binding, the ErbB receptors form homo- or hetero-dimers with subsequent phosphorylation of the tyrosine kinase domain and activation of different signalling pathways, including the RAS/MEK/ERK and the PI3K/AKT pathways [9]. EGF-like peptides are abundantly produced and secreted by human breast cancer cells [10]. Transforming growth factor α (TGFα) is one of the most potent ligands of the EGFR [10]. While EGF forms an EGF/EGFR complex that is degraded in lysosomes, the TGFα/EGFR complex favours the recycling of the receptor, thus resulting in a more potent mitogenic signal and a greater DNA synthesis compared to EGF [11, 12]. High levels of expression of TGFα have been reported in several tumor types, including breast cancer [7, 13].

Because several studies demonstrated that MSCs express a functional EGFR [14–16], we hypothesized that TGFα might be involved in the cross-talk between MSCs and breast cancer cells within the tumor microenvironment [17]. Previous studies, including observations from our group, have indeed demonstrated that treatment with TGFα or EGF increased in MSCs the production of several factors which might promote tumor growth and/or angiogenesis, such as VEGF, IL6, angiopoietin-2 (ANG-2), granulocyte-colony-stimulating factor (G-CSF), hepatocyte growth factor (HGF) and heparin-binding EGF (HB-EGF) [15, 18, 19]. However, these studies did not provide a comprehensive picture of the effects of EGFR signalling on the secretome of MSCs.

RNA sequencing (RNA-seq), using next-generation-sequencing (NGS) platforms, has greatly improved the analysis of whole transcriptome, allowing for the complete annotation and quantification of a large number genes in a single run. This technology allows to detect known and uncharacterized transcripts, and provides information on alternative and novel splicing events [20].

Using the RNA-Seq technology, we analysed the whole transcriptome of MSCs stimulated with TGFα in order to comprehensively assess the genes regulated by the EGFR signalling in MSCs. We identified a panel of growth factors, cytokines and chemokines potentially involved in the EGFR-mediated cross-talk between MSCs and breast cancer cells. These findings have increased our knowledge on the mechanisms of breast cancer progression and might allow to develop novel therapeutic strategies targeting the tumor-stroma interaction.

## RESULTS

### RNA-seq analysis

In order to characterize the whole transcriptome of MSCs following EGFR activation, we stimulated serum-starved MSCs with recombinant TGFα (10 ng/ml) for 1 hour and purified poly(A) RNA fractions from not stimulated (MSC) or TGFα-stimulated (MSC+TGFα) MSCs. Using the SOLiD 5500xl platform, we sequenced four libraries of cDNA from MSCs and four from MSC+TGFα, and an average number of reads of about 37 million for MSC and 47 million for MSC+TGFα was obtained (Supplementary table 1). Quality analysis revealed that more than 50% of sequences had a median quality score value of 31. Sequence reads were aligned to the human genome hg19, using the LifeScope software. The mapping of the sequence reads to the reference genome evidenced a mean coverage of 80% for MSC and 77% for MSC+TGFα (Supplementary table 1).

**Figure 1:**
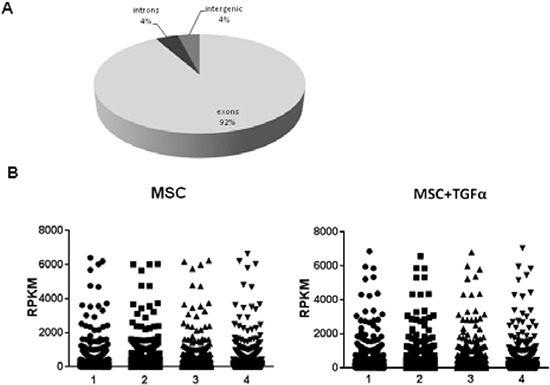
Sensitivity and reproducibility of RNA seq analysis **(A)** Distribution of mapped reads onto the reference human genome hg19. The vast majority of the reads (92%) fall onto exons, whereas the remaining fall onto introns (4%) or intergenic regions (4%). **(B)** Comparison between RPKM values obtained for untreated and TGFα-treated MSCs replicates. Statistical analysis with ANOVA test revealed a very high reproducibility within replicates with a *P*-value>0.999.

The analysis of the mean distribution of the reads onto the reference genome indicated that the majority of the reads fell onto exons (92%), while only a minority of the reads fell onto introns (4%) or intergenic regions (4%) (Figure 1A). The presence of some intronic reads in total poly(A) RNA might be due to partially processed RNAs and/or to currently unannotated internal exons [21]. To compare gene expression levels within and between replicates, Reads Per Kilobase per Million of mapped reads (RPKM) values for each gene were calculated [21]. RPKM values obtained in the four replicates of untreated and TGFα-treated MSCs resulted to be highly reproducible, as demonstrated by statistical analysis (ANOVA test, *P*-value>0.999) (Figure 1B).

**Figure 2:**
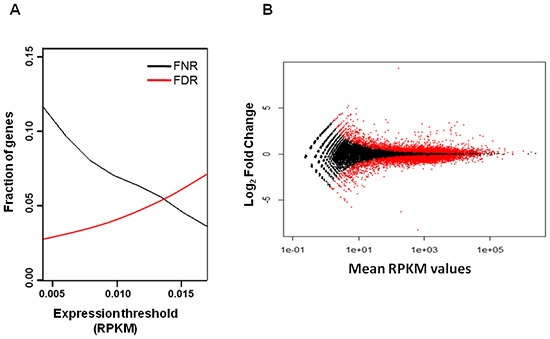
False discovery rate (FDR) and false negative rate (FNR) analysis for different RPKM values **(A)** The 0.013 RPKM threshold value corresponds approximately to 0.05 FDR and 0.05 FNR. **(B)** Scatter plot of log_2_ fold change versus mean RPKM values of genes expressed in untreated and TGFα-treated MSCs. The red dots identify 10,068 genes that are differentially expressed at *P*-value=0.05 corrected with 0.1 FDR between untreated and TGFα-treated MSCs.

We next calculated false discovery rate (FDR) and false negative rate (FNR) for different RPKM values and identified a RPKM threshold value of 0.013, that balanced the number of false negatives and false positives (Figure 2A). Applying this threshold, we identified 19,669 genes expressed in MSCs. We then selected 10,068 genes that are differentially expressed between untreated and TGFα-treated MSCs at *P*-value = 0.05 corrected with 0.1 FDR, according to the statistical analysis performed with the R-based package DESeq [22] (Figure 2B and Supplementary table 2).

For the 10,068 genes, we calculated the differential gene expression as Fold Induction (FI). We focused our attention on 1,640 genes that we considered highly differentially regulated. In particular, 967 genes were up-regulated with FI≥1.50 and 673 genes were down-regulated with FI≤0.50 (Supplementary table 3).

### Functional enrichment analysis of highly regulated genes

To investigate the biological role of the genes modulated in MSCs at transcriptional level following EGFR activation, we categorized the 1,640 highly regulated genes (FI≤0.50 and FI≥1.50) into enriched categories according to GO molecular function classification and KEGG pathways analysis.

The top 6 GO categories that resulted significantly enriched with a *P* value <0.01 were: growth factor activity; cytokine activity; protein kinase regulator activity; structural molecule activity; cytokine receptor activity; and kinase regulator activity. The complete list of genes included in each category has been reported in Table 1. TGFα-treated MSCs resulted to be enriched in genes coding for members of different families of growth factors: VEGF (*VEGFA* and *FIGF*/*VEGFD*); fibroblast growth factor (*FGF10* and *FGF17*); TNF ligands superfamily (*NGF, CD70, TNFSF13B, TNFSF4* and *TNFSF9*); EGF-like proteins (*HB-EGF*, *EREG* and *NRG1*) and their receptors (*ERBB3*)*;* and other growth factors (*LIF* and *KITLG/SCF*). Enriched categories also included several chemokines (*CCL19*, *CCL2*, *CCL25* and *CXCL3)*, interleukins (*IL6, IL15*, *IL17B*, *IL19* and *IL1B)* and interleukin receptors (*IL12RB2*, *IL18R1*, *IL20RA*, *IL9R, IL1R2*, *IL12RB1*, *IL2RB* and *IL7R)*. Moreover, EGFR activation in MSCs produced a significant enrichment in genes belonging to the TGFβ superfamily (*BMP3*, *INHBA*, *INHBC, INHBE, MSTN, GDF15* and *TGFB3*), that plays a role in cell migration, invasion and epithelial-mesenchymal transition (EMT).

**Table 1:** Gene ontology (GO) enrichment analysis of the 1,640 genes highly regulated in untreated and TGFα-treated MSCs

GO Term	Count	P-Value	Down-regulated genes (FI≤0.50)^§^	Up-regulated genes (FI≥1.50)^§^
**Growth factor activity**	33	1.43E-08	***BMP3**, **ENDOU**, **FIGF**, GMFB, **KITLG**, **MSTN**, **NDP**, **OGN**, **TFF1***	***CLCF1**, **EREG**, **FGF10**, **FGF17**, **GDF15**, **GDNF**, GMFG, **HB-EGF**, **IL1B**, **IL6**, **INHBA**, **INHBC**, **INHBE**, KGFLP1, **LEFTY2**, **LIF**, **MIA**, **NGF**, **NRG1**, **OSGIN1**, **PSPN**, **TGFB3**, **THPO**, **VEGFA***
**Cytokine activity**	27	4.54E-04	***ADIPOQ**, **BMP3**, CD70, IFNE, **IL15**, **IL17B**, **IL19**, **MSTN**, **PF4**, **TNFSF13B**, **TNFSF4***	***CCL19**, **CCL2**, **CCL25**, **CLCF1**, CMTM1, **CXCL3**, **EBI3**, **GDF15**, **IL1B**, **IL6**, **INHBA**, **LEFTY2**, **LIF**, **THPO**, **TNFSF9**, **VEGFA***
**Protein kinase regulator activity**	14	0.002	*AGAP2, CCNE2, GMFB, PKIB*	*C1orf230, CDK5R1, CDKN1A, ERBB3, GMFG, **NRG1**, PPP1R1B, RAPGEF4, SOCS3, TRIB1*
**Structural molecule activity**	61	0.003	*CCIN, CRYGS, **KAL1**, KRT15, MPZ, MRPL13, MRPS10, OCLN, RPL35A, TNXA, **WNT16***	*CAV3, CLDN2, CLDN20, CLDN4, CLDN5, **COL27A1**, **COL4A3**, **CRYBB2**, FLG, HOMER2, IMPG2, KRT14, KRT17, KRT18, KRT24, KRT32, KRT34, KRTAP1-1, KRTAP1-3, **LAD1**, **MUC2**, **MUC5B**, MYH11, MYL2, NEB, PPL, RPL12, RPL14, RPL19, RPL23A, RPL27, RPL32, RPS11, RPS14, RPS15, RPS19, RPS27, SPTA1, SPTB, SPTBN4, TUBA3E, TUBB2A, TUBB2C, TUBB3, TUBB4Q, TUBBP5, TUBG1, UPK1B, **WNT2**, **WNT3***
**Cytokine receptor activity**	11	0.003	*CSF2RB, IL12RB2, IL18R1, IL20RA, IL9R*	***EBI3**, CSF3R, IL12RB1, IL1R2, IL2RB, IL7R*
**Kinase regulator activity**	15	0.003	*GMFB, AGAP2, CCNE2, MOBKL1A, PKIB*	*C1orf230, CDK5R1, CDKN1A, ERBB3, GMFG, **NRG1**, PPP1R1B, RAPGEF4, SOCS3, TRIB1*

§ Genes coding for secreted or potentially secreted proteins are indicated in bold; genes coding for receptors are underlined (according to UniProtKB classification).

Transcripts coding for proteins associated with cellular motility and cell-cell adhesion, such as cytokeratins (*KRT14*, *KRT17*, *KRT18*, *KRT24*, *KRT32*, *KRT34* and *KRT15*), tubulins (*TUBA3E*, *TUBB2A*, *TUBB2C*, *TUBB3*, *TUBB4Q* and *TUBG1*) and claudins (*CLDN2*, *CLDN20*, *CLDN4* and *CLDN5*), also resulted to be enriched in MSCs following treatment with TGFα. Finally, EGFR activation modulated the expression of genes coding for regulators of cell cycle (*CCNE2, CDK5RI* and *CDKN1A)* and of cell signalling (*ERBB3, SOCS3, TRIB1* and *AGAP2).*

Within GO enriched categories we found both up-regulated and down-regulated genes (Table 1). In this respect, we found that a large number of genes coding for potentially secreted proteins resulted up-regulated following TGFα treatment (n. 47), whereas 19 genes were down-regulated. Among the up-regulated genes, we found several genes that have been already shown to have a role in the pathogenesis of breast cancer and to be potentially involved in the interaction between MSCs and breast cancer cells, including *VEGFA, IL6, EREG, HB-EGF, LIF, NGF, NRG1, CCL19, CCL2, CCL25* and *CXCL3* (Table 1).

To evaluate the enrichment in signalling pathways, we performed KEGG analysis on the 1,640 highly regulated genes. The top 3 most enriched pathways were cytokine-cytokine receptor interaction, Jak-STAT and MAPK signalling pathway (*P* value <0.01) (Table 2). The list of genes included in this analysis significantly overlapped with the list of the enriched GO categories, with particular regard to secreted proteins and cell membrane receptors. Importantly, TGFα stimulation also produced in MSCs a significant up-regulation of genes that are known to be induced by EGFR activation, such as the transcription factors *FOS*, *JUN, JUND* and *ELK4,* and different inhibitors of MAPK kinase activity belonging to Sprouty (*SPRY2* and *SPRY4*) and DUSP (*DUSP1, DUSP2, DUSP5, DUSP6* and *DUSP8*) families. Finally, KEGG pathways analysis confirmed up-regulation of genes coding for VEGF and NGF, and revealed up-regulation of the expression of their cognate receptors, *KDR* and *NTRK1*, respectively.

**Table 2:** KEGG pathways analysis of the 1,640 genes highly regulated in untreated and TGFα-treated MSCs

Pathway	Count	P-Value	Down-regulated genes (FI≤0.50)^§^	Up-regulated genes (FI≥1.50)^§^
**Cytokine-cytokine receptor interaction**	42	1.40E-07	*CD70, CSF2RB, **FIGF**, FLT1, IFNE, IL12RB2, **IL15**, **IL17B**, IL18R1, **IL19**, IL20RA, IL23R, IL9R, KIT, **KITLG**, **PF4**, **TNFSF13B**, **TNFSF4***	***CCL2**, **CCL25**, **CLCF1**, CSF3R, **CXCL3**, IL12RB1, **IL1B**, IL1R2, IL2RB, **IL6**, IL7R, **INHBA**, **INHBC**, **INHBE**, KDR, **LIF**, **TGFB3**, TNFRSF10A, TNFRSF10C, TNFRSF12A, TNFRSF17, **TNFSF9**, **VEGFA**, **CCL19***
**Jak-STAT signaling pathway**	24	2.05E-04	*CSF2RB, IFNE, IL12RB2, **IL15**, **IL19**, IL20RA, IL23R, IL9R, SPRY3*	*CSF3R, IL12RB1, IL2RB, **IL6**, IL7R, **LIF**, MYC, PIM1, PTPN6, SOCS3, SOCS7, SPRY2, SPRY4, **CLCF1***
**MAPK signaling pathway**	30	5.62E-03	*CACNA2D3, RAP1A, RASGRP3*	*ARRB1, DDIT3, DUSP1, DUSP2, DUSP5, DUSP6, DUSP8, ELK4, **FGF10**, **FGF17**, FOS, GADD45B, HSPA1L, L1B, IL1R2, JUN, JUND, MAP2K3, MAP3K13, MAP3K14, MYC, NGF, NR4A1, NTRK1, PLA2G2A, PRKACG, **TGFB3***

§ Genes coding for secreted or potentially secreted proteins are indicated in bold; genes coding for receptors are underlined (according to UniProtKB classification).

### Effects of TGFα treatment on protein secretion in MSCs

Because EGFR activation in MSCs significantly affected the expression of genes coding for secreted proteins, we focused on selected factors to assess whether transcriptional regulation resulted in increased protein secretion.

**Figure 3:**
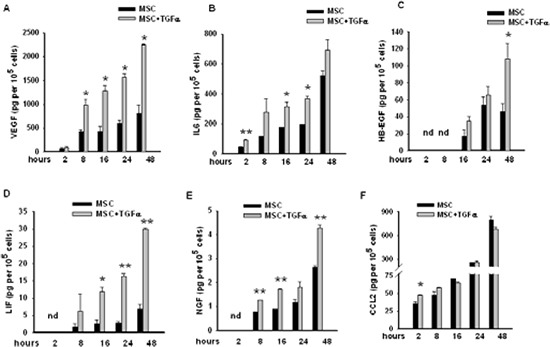
Levels of a panel of secreted factors in conditioned media from untreated and TGFα-treated MSCs The levels of **(A)** VEGF, **(B)** IL6, **(C)** HB-EGF, **(D)** LIF, **(E)** NGF and **(F)** CCL2 were assessed using Bio-plex Cytokines Arrays (mean ±S.D., *P < 0.05, ** *P* < 0.005 for comparison between untreated versus TGFα-treated MSCs at the different time points, two-tailed Student's *t*-test).

We found that EGFR signalling significantly up-regulated the transcription of *VEGFA* (FI=2.01) and *IL6* (FI=10.47) (Supplementary table 3). Previous data from our group have indeed demonstrated that treatment of MSCs for 96 hours with TGFα leads to a significant increase in the secretion of both VEGF and IL6 [15]. In order to evaluate the effects of a brief exposure to TGFα on the secretion of these factors in MSCs, we performed time course experiments using the xMAP Bio-Plex Cytokine array system. Time course analysis revealed that TGFα induced in MSCs the release of VEGF and IL6 as early as 2 to 8 hours after the start of treatment (Figure 3A and 3B).

We next investigated whether transcriptional regulation of additional secreted factors also resulted in an increased protein release in MSCs following TGFα treatment. For this purpose, we measured the levels of HB-EGF, LIF, NGF and CCL2 in the conditioned media from untreated or TGFα-treated MSCs at different time points. Accordingly with RNA-seq data demonstrating that treatment of MSCs with TGFα induced the transcription of *HB-EGF* (FI=5.60), *LIF* (FI=9.01), *NGF* (FI=1.63) and *CCL2* (FI=1.88) (Supplementary table 3), we observed an increase of the secretion of the corresponding proteins, although at different extent and with different kinetic (Figure 3). In particular, the secretion of HB-EGF, LIF and NGF gradually increased following treatment with TGFα (Figure 3C-E). Only a modest raise in the levels of CCL2 was observed in TGFα-treated MSCs at 2 and 8 hours followed by a mild reduction at the later time points (Figure 3F).

Taken together, these data confirm that TGFα stimulation induces in MSCs an early and prolonged secretion of growth factors, cytokines and chemokines that are modulated at transcriptional level.

### Expression of alternative splicing variants of VEGFA in MSCs

Alternative splicing significantly increases functional gene diversity, and aberrant splicing has been shown to contribute to tumor progression and cancer therapy resistance [23]. To evaluate whether EGFR activation regulated in MSCs the expression of alternatively spliced genes, the RNA-seq data were analysed using the Splice Finding tool. Among the 19,669 genes expressed in MSCs, we identified 4,377 and 4,262 alternatively spliced genes in untreated and TGFα-treated MSCs, respectively. We focused our attention on selected genes coding for secreted factors listed in tables 1 and 2. For all the investigated genes we found only one mature transcript, except for *VEGFA, GDNF, NRG1* and *OGN* (data not shown).

**Figure 4:**
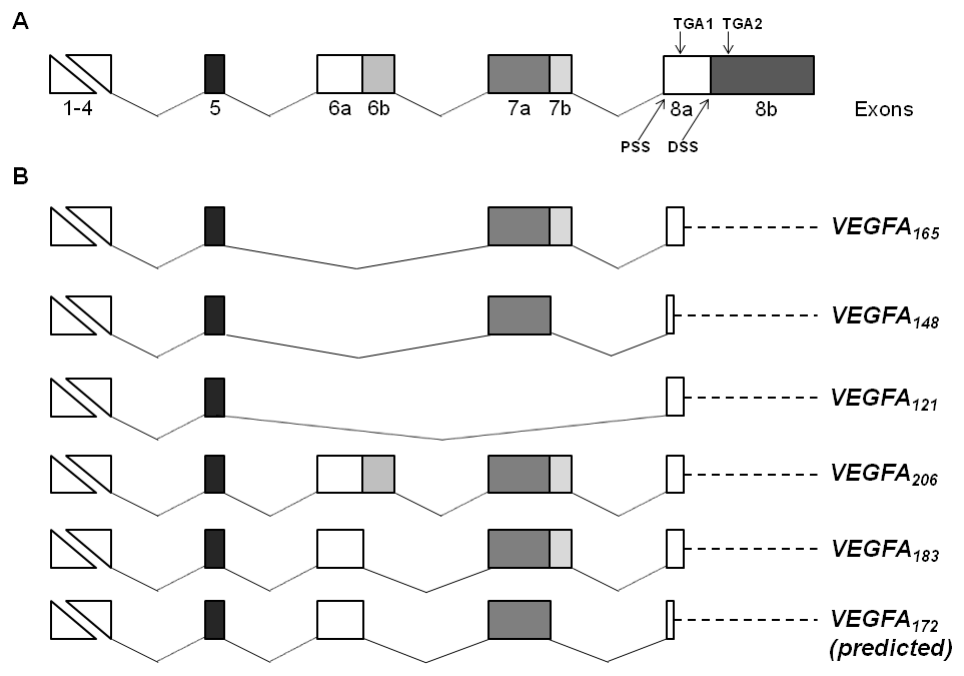
Schematic representation of *VEGF* gene structure and splice isoforms identified in MSCs **(A)** Structure of human *VEGF* gene. The *VEGF* gene consists of eight exons, and exons 6, 7 and 8 are composed of parts “a” and “b”. Proximal splice site (PSS), distal splice site (DSS) and alternative stop codons (TGA1 and TGA2) in exon 8 are indicated. **(B)** Alternative splice variants of *VEGF* gene expressed in untreated and/or TGFα-treated MSCs. VEGF variants are named according to the amino acid number of the mature proteins. The length of the novel VEGFA_172_ isoform has been predicted using the Translate tool. In the VEGFA_165_, VEGFA_121_, VEGFA_206_ and VEGFA_183_ isoforms the translation ends at the first stop codon (TGA1). For VEGFA_148_, and putatively for VEGFA_172,_ a premature stop codon is formed by the out of frame fusion of exons 7a and 8a, resulting in a truncated protein. Dashed lines identify 3′untraslated regions.

In particular, *VEGFA* showed 6 different splice isoforms that differed for the presence or absence of the exons 6a, 6b and 7b (Figure 4). Five variants encoded for the already characterized isoforms VEGFA_165_, VEGFA_148_, VEGFA_121_, VEGFA_206_ and VEGFA_183_. The other isoform was an unannotated splice variant of *VEGFA* of 3,575 bp. This variant derived from alternative 5′ splice site selection in the exons 6 and 7, resulting in the lack of the exons 6b and 7b (Figure 4). Using the Translate tool, we predicted that the novel splice variant encoded for a protein of 198 aa including the N-terminal signal peptide of 26 aa, that we named VEGFA_172_. Compared to VEGFA_206_, the predicted VEGFA_172_ lacked 17 aa encoded by exon 6b, and 17 aa at the C terminal of the protein. The fusion between 7a and 8a resulted in the substitution of an alanine by a methionine and in a premature stop codon, similarly to the VEGFA_148_ isoform (Supplementary figure 1). BLAST analysis of nucleotide and protein sequences revealed that the novel isoform does not match with any sequence in the respective databases. We identified the *VEGFA_165_, VEGFA_148_*, *VEGFA_121_* and *VEGFA_206_* transcripts both in untreated and TGFα-treated MSCs, whereas the *VEGFA_183_* and *VEGFA_172_* variants were found only in MSCs stimulated with TGFα, thus suggesting that TGFα affects quality and quantity of VEGF transcription.

**Figure 5:**
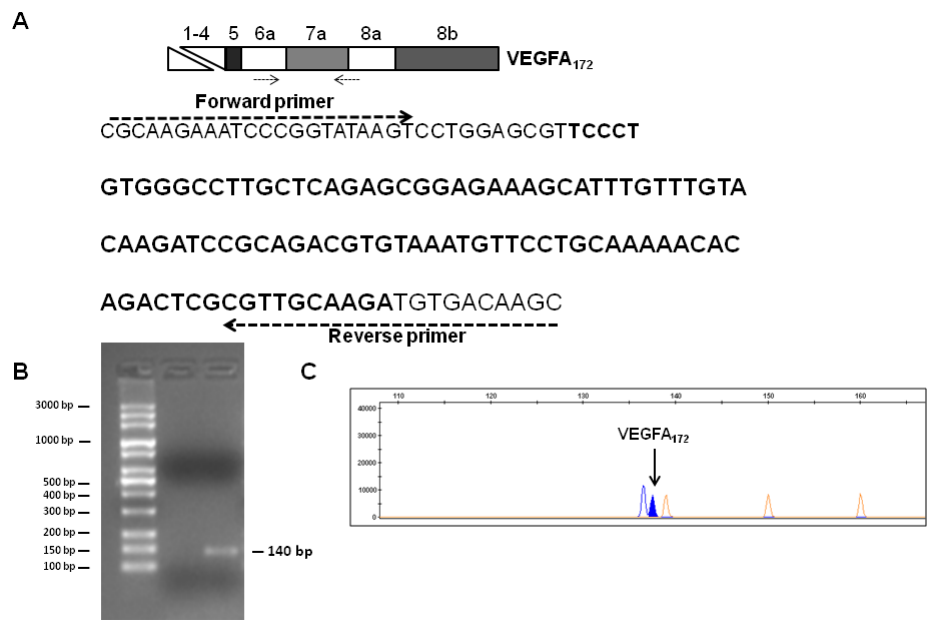
Analysis of *VEGFA_172_* expression in TGFα-treated MSCs **(A)** Sequence of the PCR product (140 bp) obtained by RT-PCR analysis of *VEGFA_172_* in TGFα-treated MSCs. The positions of forward and reverse primers are shown. The sequence of exon 7a is reported in bold. Agarose gel electrophoresis **(B)** and fragment analysis **(C)** of the RT-PCR product for *VEGFA_172_* in TGFα-treated MSCs.

To confirm the expression of the *VEGFA_172_* variant in TGFα-treated MSCs, we performed RT-PCR analysis using a forward PCR primer located on exon 6a and a reverse PCR primer spanning exons 7a/8 boundaries (Figure 5A). Agarose gel electrophoresis and fragment analysis of the PCR product confirmed the expression of the *VEGFA_172_* transcript in TGFα-treated MSCs (Figure 5B and 5C). The sequence of the new VEGF splice isoform was also verified by direct sequencing of the PCR product (data not shown).

## DISCUSSION

Using RNA-seq, we provided for the first time a comprehensive assessment of the genes regulated in MSCs by EGFR signalling. A previous study evaluated the gene expression profile of epithelial MCF10A and HeLa cell lines stimulated with EGF for 1 hour using a microarray approach [24]. Even though different technical approaches (microarray versus RNAseq), growth factors (EGF versus TGFα) and cell types (epithelial versus mesenchymal) have been used, 271 and 134 genes induced by EGF, respectively, in HeLa and MCF10A cells resulted differentially expressed also in MSCs following TGFα stimulation. In particular, EGFR activation induced both in epithelial cells and in MSCs the up-regulation of the immediate early response transcription factors *JUN*, *FOS* and *EGR1*, and of several negative regulators of transcription such as *FOSL1*, *JUNB*, *KLF6* and *MAFF,* that are delayed response genes implicated in negative transcriptional regulation of the immediately early response genes [24]. Finally, genes coding for *ZFP36,* that has been described to induce mRNA degradation, and for DUSP proteins (*DUSP4,DUSP6*), that dephosphorylate MAPK, were up-regulated both in MSCs and in epithelial cells, following EGFR activation [24, 25]. In agreement with data from Amit and colleagues, our results strongly confirm that the EGFR was activated in response to TGFα in MSCs and revealed that a significant number of genes involved in EGFR signalling regulation are in common between epithelial cells and MSCs.

Our data demonstrate that EGFR signalling produces significant modifications in the secretome of MSCs. Indeed, GO and KEGG analyses showed that TGFα produced in MSCs a significant enrichment in genes coding for growth factors, cytokines and chemokines and in pathways associated with growth factors and cytokines, such as MAPK and Jak-STAT pathways [9, 26]. In particular, TGFα treatment induced in MSCs the expression of the angiogenic factors *VEGFA* and *IL6* that, in agreement with our previous findings, was also confirmed at protein level. In this regard, we have previously described that TGFα treatment of MSC for 96 hours also induced an increase in the secretion of other pro-angiogenic factors such as IL8, leptin and PECAM1 [15]. As shown in Supplementary table 3, *PECAM1* gene expression was significantly up-regulated (FI=2.32) following treatment of MSCs with TGFα for 1 hour. In contrast, a marginal although statistically significant increase in the expression of *IL8* (FI=1.18) and *LEP* (the leptin gene, FI=1.28) was found (Supplementary table 2). These latter findings might indicate that prolonged stimulation with TGFα or post-transcriptional mechanisms are required for IL8 and leptin EGFR-induced secretion [15].

Among the cytokines and chemokines, we found that TGFα significantly modulated LIF that has been demonstrated to be associated to breast cancer transformation and progression [27, 28]. We also found a marginal increase in the levels of secretion of CCL2, which has been shown to stimulate the migration of breast cancer cells [3]. Furthermore, TGFα induced in MSCs a significant increase in the expression of growth factors such as NGF and HB-EGF that was confirmed at protein level as well. NGF has been reported to promote breast cancer cell proliferation and invasion [29]. Intriguingly, a recent study demonstrated that EGF induced in MSCs an autocrine loop mediated by EGR1 and involving the EGFR ligands HB-EGF and AREG. This autocrine circuit was found to stimulate the secretion by MSCs of other growth factors and cytokines, including VEGF, LIF, IL6 and IL11 [19]. In agreement with these data, we found in our study a significant increase in the expression of HB-EGF, VEGF, IL6, LIF and EGR1 (FI=3.82; Supplementary table 3) in TGFα-treated MSCs as compared with untreated cells. We also demonstrated that EGFR stimulation up-regulated in MSCs the expression of different members of the TGFβ superfamily. Because it has been demonstrated that TGFβ secreted by cancer-associated fibroblasts promotes EMT in breast cancer cells [30] as well as in other tumor types and EMT has a fundamental role in the metastatic spreading, MSC-derived TGFβ-like factors might play an important role in cancer progression. Finally, it must be underlined that TGFα was also found to induce the expression of several receptors of growth factors, cytokines and chemokines including ERBB3 (FI=1.54; Supplementary table 3), which might enhance the above mentioned autocrine loop by forming heterodimers with the EGFR. Taken together, these data suggest that EGFR activation induces a cascade of events leading to an increase of the ability of MSCs to both release factors favouring tumor progression and respond to signals involved in the cross-talk with cancer cells within the tumor microenvironment.

RNA-seq offers the possibility of identifying unknown transcripts [20, 31, 32]. In this regard, we found that in MSCs several genes coding for secreted proteins have alternatively spliced variants, including *VEGFA*, *NRG1*, *GDNF* and *OGN*. Importantly, we also demonstrated that TGFα-treated MSCs express a novel splice variant of VEGF. The VEGF gene encodes for different isoforms with distinct biological properties, generated by skipping of exon 5 and alternative splice site selection of exons 6, 7 and 8. VEGF isoforms differ in their heparin binding ability as well as in receptor affinity [33]. The novel isoform that we identified lacks exons 6b and 7b and potentially codes for a protein of 198 aa. Since exons 6 and 7 encode for the heparin binding domain of VEGF, we hypothesize that in the predicted *VEGFA_172_* isoform the heparin binding affinity could be affected. VEGF binds to two receptors, vascular endothelial growth factor receptor (VEGFR)-1 and -2, and two co-receptors, neuropilin (NRP)-1 and 2. It has been suggested that exons 7 and 8a are required for binding to NRP-1, while exon 6 and 8a sequences cooperate in NRP-2 binding [34]. VEGFA_172_ lacks 17 residues encoded by exon 6b, and 17 residues at the C terminal of the protein corresponding to exons 7b and 8a. Preliminary structural analysis suggest that VEGFA_172_ might not be able to bind to NRP-1 but should interact with NRP-2. However, further structural and functional studies will be necessary to confirm this hypothesis. The biological role of the different isoforms is not fully clarified. The selection of the proximal splice site in exon 8a results in the generation of pro-angiogenic forms of VEGF, whereas the selection of the distal splice site in exon 8b produces anti-angiogenic forms of VEGF, so that the balance of the different isoforms can either promote or inhibit angiogenesis [35]. Interestingly, we identified in MSCs different pro-angiogenic isoforms of *VEGFA,* but not anti-angiogenic variants. In this regard, as the distal splice site in exon 8b has been selected in *VEGFA_172_* transcript, the resulting protein could also probably belong to the pro-angiogenic family of VEGF isoforms. It is possible that different pro-angiogenic isoforms have a different biological activity. For example, VEGFA_121_ has been reported to have a stronger angiogenic activity than VEGFA_165_ in breast cancer [36]. On the other hand, lack of binding to NRP-1 might reduce the angiogenic power of VEGFA_172_ [35]. Future investigations on the functional activity of the new VEGF isoform are ultimately required.

Our findings might have potential clinical implications. Although the EGFR and its ligands are frequently expressed in breast carcinoma, anti-EGFR agents, such as cetuximab, panitumumab, gefitinib and erlotinib, have produced disappointing results in breast cancer [37]. Our results might suggest that EGFR signalling induces within the tumor microenvironment the release by MSCs of soluble factors that might sustain breast cancer cell growth through different signalling pathways that may also be responsible of resistance to anti-EGFR agents. Interestingly, the factors whose secretion was found to be strongly induced in MSCs following TGFα stimulation, mainly signal through the MAPK and Jak-STAT pathways. Different STAT3 and Jak inhibitors are currently in clinical trials and several agents targeting the MAPK signalling pathway have been tested clinically or are currently undergoing clinical trial evaluation [38, 39]. Because it has been recently highlighted the importance of targeting different mechanisms in the tumor microenvironment using combination of drugs [40], we can speculate that anti-EGFR drugs in combination with anti-MAPK signalling agents or Jak-STAT inhibitors might more efficiently block the interaction between MSCs and breast cancer cells.

In conclusion, our data demonstrate that EGFR activation leads to a significant change in the expression of a wide array of genes coding for secreted proteins that can significantly enhance tumor progression by acting on several mechanisms within the tumor microenvironment. Interestingly, EGFR signalling was found to induce both quantitative and qualitative changes in the secretome of MSCs, as also demonstrated by the finding of a novel *VEGFA* splice variant in TGFα-treated MSCs. Taken together, our data suggest that a better understanding of the factors and the mechanisms involved in the MSCs-breast cancer cells cross-talk might provide the rationale to develop novel therapeutic strategies aimed to inhibit breast cancer progression.

## METHODS

### Cell line

Bone marrow-derived MSCs were purchased from Lonza (Verviers, Belgium) and maintained in MSCGM bullet kit (Lonza) in a humidified atmosphere at 37°C and 5% CO_2_, as suggested by the provider. MSCs were positive for CD29, CD44, CD105, CD166 and negative for the markers of the hematopoietic lineage CD14, CD34 and CD45. Cells were used at passages 3–4.

### RNA isolation

MSCs were starved overnight in serum free medium and treated for 1 hour with recombinant human TGFα (PeproTech,Rocky Hill, NJ, USA) at a concentration of 10 ng/ml. Total RNA was extracted from untreated or TGFα-treated MSCs using TRIReagent, according to the manufacturer's protocol (Ambion/Life Technologies, Milan, Italy). Poly(A) RNA was isolated using the Ambion MicroPoly(A) Purist Kit (LifeTechnologies).

### Whole transcriptome libraries preparation and RNA-seq

Poly(A) RNA samples were fragmented using RNASE III and the SOLiD Total RNA-Seq Kit (Life Technologies). Following cleanup, fragments with an average size between 125 and 200 nucleotides were obtained, as determined using the Agilent 2100 Bioanalyzer and the RNA 6000 Pico Kit (Agilent Technologies, Milan, Italy). Fragmented RNA was subjected to hybridization and ligation to SOLiD adaptor mix. cDNA libraries were subsequently generated by reverse transcription and purified using the Agencourt AMPure XP Kit (Beckman Coulter). Purified cDNA was amplified using SOLiD 5′PCR primers and barcoded SOLiD 3′PCR primers using the SOLiD RNA Barcoding Kit (Life Technologies), in order to prepare cDNA libraries for multiplex sequencing. Amplified cDNA was purified using the PureLink PCR Micro Kit (Invitrogen/Life Technologies) and quantified by Qubit (Invitrogen/Life Technologies). The average size (224 bp) of the cDNA fragments was determined using the Agilent 2100 Bioanalyzer and the High Sensitivity DNA Kit (Agilent Technologies). Barcoded cDNA libraries were captured to the surface of beads, amplified by emulsion PCR and enriched using the SOLiD EZ Beads System (Life Technologies). Beads were deposited onto a glass slide and sequenced on the Applied Biosystems SOLiD 5500xl platform (LifeTechnologies) using the paired-end protocol (75 bp + 35 bp).

### Sequencing data analysis

The analysis of the quality of raw data was performed using the Galaxy platform (http://galaxyproject.org). Whole-transcriptome reads were aligned to the version 19 of the human genome (hg19) with the SOLiD LifeScope Genomic Analysis Software version 2.5 (Life Technologies) using the parameters recommended in the user's manual. The automatic quantification of transcriptional events across the entire genome was performed with LifeScope. The number of observed counts (number of reads/gene) was normalized for the length of the transcript and the number of mapped reads (RPKM) (Reads Per Kilobase per Million of mapped reads). Comparison between RPKM values obtained for untreated and TGFα-treated MSCs replicates was performed by ANOVA test based on one-way analysis of variance where *P*-value<0.05 is considered as index of statistically significant difference. Statistical evaluation of differential gene expression between untreated and TGFα-treated MSCs was assessed by DEseq tool in R package that plots log_2_ fold change versus normalized counts and uses a significance level *P*-value=0.05 corrected with a FDR test threshold of 0.1 [22].

The full dataset of raw data has been deposited in the GEO database (accession number: GSE60560).

### Splice variant analysis

Splice Finding tool (Lifescope software) was used to detect alternatively spliced transcripts of VEGF. The prediction of the amino acid sequence of the unannotated splice isoform of VEGF was performed using the Translate tool (http://web.expasy.org/translate).

### Gene enrichment analysis

The Database for Annotation, Visualization and Integrated Discovery (DAVID; http://david.abcc.ncifcrf.gov) was used to perform functional annotation analysis of enriched gene ontology (GO) terms and KEGG pathways. Statistical significance was evaluated with a modified Fisher's exact test (EASE score) and GO and KEGG terms with P values < 0.01 were considered significant.

### Preparation of conditioned media and immunoassays

MSCs were seeded in 48-well cell culture plates (18×10^3^ cells/well) and serum starved overnight. Then, cells were treated with TGFα (10 ng/ml) and conditioned media were collected at 2, 8, 16, 24 and 48 hours after treatment, filtered with 0.22 μm syringe filters and stored in aliquots at −80°C.

The concentration of VEGF, IL6, HB-EGF, LIF, NGF)and CCL2 in the conditioned media from untreated and TGFα-treated MSCs was determined using Bio-Plex Cytokine Arrays, according to the manufacturer's protocol (Bio-Rad Life Science, Milan, Italy). The levels of secreted proteins in conditioned media were referred as picograms per 10^5^ cells, as determined on the harvesting time.

Statistical significance was determined using two-tailed Student's *t*-test. *P* values < 0.05 were considered significant.

### RT-PCR and Length Analysis of Fluorescently Labelled PCR Products (Fragment Analysis)

cDNA synthesis was performed with SuperScript II Reverse Transcriptase (Life Technologies) using random hexamers and 2 μg of total RNA. PCR amplification was performed using the following FAM-labelled forward primer 5′-CGCAAGAAATCCCGGTATAA-3′ and the reverse primer 5′-GCTTGTCACATCTTGCAACG-3′. PCR was performed using 1X AmpliTaq Gold DNA Polymerase Buffer (Applied Biosystems); 2mM MgCl_2_, 0.2mM dNTPs, 0.2μM of each primer and 2.5 U of AmpliTaq Gold DNA Polymerase (Applied Biosystems). Thermocycler conditions were as follows: 95°C for 10 min, 40 cycles of 95°C for 1 min, 58°C for 1 min, 72°C for 1.5 min and a final extension step of 10 min at 72°C. The PCR product was analysed by 3% agarose gel electrophoresis and with four-color laser-induced fluorescence capillary electrophoresis system (3500 DX Genetic Analyzer, Life Technologies). Data were evaluated with the GeneMapper 4.1v Analysis Software (Life Technologies).

## SUPPLEMENTARY FIGURE AND TABLES







## References

[1] El-Haibi CP, Karnoub AE (2010). Mesenchymal stem cells in the pathogenesis and therapy of breast cancer. J Mammary Gland Biol Neoplasia.

[2] Karnoub AE, Dash AB, Vo AP, Sullivan A, Brooks MW, Bell GW, Richardson AL, Polyak K, Tubo R, Weinberg RA (2007). Mesenchymal stem cells within tumour stroma promote breast cancer metastasis. Nature.

[3] Molloy AP, Martin FT, Dwyer RM, Griffin TP, Murphy M, Barry FP, O'Brien T, Kerin MJ (2009). Mesenchymal stem cell secretion of chemokines during differentiation into osteoblasts, and their potential role in mediating interactions with breast cancer cells. Int J Cancer.

[4] Goldstein RH, Reagan MR, Anderson K, Kaplan DL, Rosenblatt M (2010). Human bone marrow-derived MSCs can home to orthotopic breast cancer tumors and promote bone metastasis. Cancer Res.

[5] Chaturvedi P, Gilkes DM, Wong CC, Luo W, Zhang H, Wei H, Takano N, Schito L, Levchenko A, Semenza GL (2013). Hypoxia-inducible factor-dependent breast cancer-mesenchymal stem cell bidirectional signaling promotes metastasis. J Clin Invest.

[6] De Luca A, Lamura L, Gallo M, Maffia V, Normanno N (2012). Mesenchymal stem cell-derived interleukin-6 and vascular endothelial growth factor promote breast cancer cell migration. J Cell Biochem.

[7] Normanno N, Bianco C, Strizzi L, Mancino M, Maiello MR, De Luca A, Caponigro F, Salomon DS (2005). The ErbB receptors and their ligands in cancer: an overview. Curr Drug Targets.

[8] Citri A, Yarden Y (2006). EGF-ERBB signalling: towards the systems level. Nat Rev Mol Cell Biol.

[9] Normanno N, De Luca A, Bianco C, Strizzi L, Mancino M, Maiello MR, Carotenuto A, De Feo G, Caponigro F, Salomon DS (2006). Epidermal growth factor receptor (EGFR) signaling in cancer. Gene.

[10] Normanno N, Bianco C, De Luca A, Salomon DS (2001). The role of EGF-related peptides in tumor growth. Front Biosci.

[11] Waterman H, Sabanai I, Geiger B, Yarden Y (1998). Alternative intracellular routing of ErbB receptors may determine signaling potency. J Biol Chem.

[12] Wilson KJ, Mill C, Lambert S, Buchman J, Wilson TR, Hernandez-Gordillo V, Gallo RM, Ades LM, Settleman J, Riese DJ (2012). EGFR ligands exhibit functional differences in models of paracrine and autocrine signaling. Growth Factors.

[13] McIntyre E, Blackburn E, Brown PJ, Johnson CG, Gullick WJ (2010). The complete family of epidermal growth factor receptors and their ligands are co-ordinately expressed in breast cancer. Breast Cancer Res Treat.

[14] Satomura K, Derubeis AR, Fedarko NS, Ibaraki-O'Connor K, Kuznetsov SA, Rowe DW, Young MF, Gehron Robey P (1998). Receptor tyrosine kinase expression in human bone marrow stromal cells. J Cell Physiol.

[15] De Luca A, Gallo M, Aldinucci D, Ribatti D, Lamura L, D'Alessio A, De Filippi R, Pinto A, Normanno N (2011). Role of the EGFR ligand/receptor system in the secretion of angiogenic factors in mesenchymal stem cells. J Cell Physiol.

[16] Normanno N, De Luca A, Aldinucci D, Maiello MR, Mancino M, D'Antonio A, De Filippi R, Pinto A (2005). Gefitinib inhibits the ability of human bone marrow stromal cells to induce osteoclast differentiation: implications for the pathogenesis and treatment of bone metastasis. Endocr Relat Cancer.

[17] De Luca A, Carotenuto A, Rachiglio A, Gallo M, Maiello MR, Aldinucci D, Pinto A, Normanno N (2008). The role of the EGFR signaling in tumor microenvironment. J Cell Physiol.

[18] Wang Y, Weil BR, Herrmann JL, Abarbanell AM, Tan J, Markel TA, Kelly ML, Meldrum DR (2009). MEK, p38, and PI-3K mediate cross talk between EGFR and TNFR in enhancing hepatocyte growth factor production from human mesenchymal stem cells. Am J Physiol Cell Physiol.

[19] Kerpedjieva SS, Kim DS, Barbeau DJ, Tamama K (2012). EGFR ligands drive multipotential stromal cells to produce multiple growth factors and cytokines via early growth response-1. Stem Cells Dev.

[20] Wang Z, Gerstein M, Snyder M (2009). RNA-Seq: a revolutionary tool for transcriptomics. Nat Rev Genet.

[21] Mortazavi A, Williams BA, McCue K, Schaeffer L, Wold B (2008). Mapping and quantifying mammalian transcriptomes by RNA-Seq. Nat Methods.

[22] Anders S, Huber W (2010). Differential expression analysis for sequence count data. Genome Biol.

[23] Oltean S, Bates DO (2013). Hallmarks of alternative splicing in cancer. Oncogene.

[24] Amit I, Citri A, Shay T, Lu Y, Katz M, Zhang F, Tarcic G, Siwak D, Lahad J, Jacob-Hirsch J, Amariglio N, Vaisman N, Segal E, Rechavi G, Alon U, Mills GB (2007). A module of negative feedback regulators defines growth factor signaling. Nat Genet.

[25] Avraham R, Yarden Y (2011). Feedback regulation of EGFR signalling: decision making by early and delayed loops. Nat Rev Mol Cell Biol.

[26] Ara T, Declerck YA (2010). Interleukin-6 in bone metastasis and cancer progression. Eur J Cancer.

[27] Dhingra K, Sahin A, Emami K, Hortobagyi GN, Estrov Z (1998). Expression of leukemia inhibitory factor and its receptor in breast cancer: a potential autocrine and paracrine growth regulatory mechanism. Breast Cancer Res Treat.

[28] Rhee DK, Park SH, Jang YK (2008). Molecular signatures associated with transformation and progression to breast cancer in the isogenic MCF10 model. Genomics.

[29] Dolle L, El Yazidi-Belkoura I, Adriaenssens E, Nurcombe V, Hondermarck H (2003). Nerve growth factor overexpression and autocrine loop in breast cancer cells. Oncogene.

[30] Yu Y, Xiao CH, Tan LD, Wang QS, Li XQ, Feng YM (2014). Cancer-associated fibroblasts induce epithelial-mesenchymal transition of breast cancer cells through paracrine TGF-beta signalling. British journal of cancer.

[31] Marioni JC, Mason CE, Mane SM, Stephens M, Gilad Y (2008). RNA-seq: an assessment of technical reproducibility and comparison with gene expression arrays. Genome Res.

[32] Martin JA, Wang Z (2011). Next-generation transcriptome assembly. Nat Rev Genet.

[33] Arcondeguy T, Lacazette E, Millevoi S, Prats H, Touriol C (2013). VEGF-A mRNA processing, stability and translation: a paradigm for intricate regulation of gene expression at the post-transcriptional level. Nucleic Acids Res.

[34] Grunewald FS, Prota AE, Giese A, Ballmer-Hofer K (2010). Structure-function analysis of VEGF receptor activation and the role of coreceptors in angiogenic signaling. Biochim Biophys Acta.

[35] Harper SJ, Bates DO (2008). VEGF-A splicing: the key to anti-angiogenic therapeutics?. Nat Rev Cancer.

[36] Zhang HT, Scott PA, Morbidelli L, Peak S, Moore J, Turley H, Harris AL, Ziche M, Bicknell R (2000). The 121 amino acid isoform of vascular endothelial growth factor is more strongly tumorigenic than other splice variants in vivo. Br J Cancer.

[37] Masuda H, Zhang D, Bartholomeusz C, Doihara H, Hortobagyi GN, Ueno NT (2012). Role of epidermal growth factor receptor in breast cancer. Breast Cancer Res Treat.

[38] Sansone P, Bromberg J (2012). Targeting the interleukin-6/Jak/stat pathway in human malignancies. J Clin Oncol.

[39] Santarpia L, Lippman SM, El-Naggar AK (2012). Targeting the MAPK-RAS-RAF signaling pathway in cancer therapy. Expert Opin Ther Targets.

[40] Swartz MA, Iida N, Roberts EW, Sangaletti S, Wong MH, Yull FE, Coussens LM, Declerck YA (2012). Tumor Microenvironment Complexity: Emerging Roles in Cancer Therapy. Cancer Res.

