# Supplementary material for: RNA-seq analysis reveals significant effects of EGFR signalling on the secretome of mesenchymal stem cells
Source: Oncotarget. 2014 Oct 29;5(21):10518–28. doi: 10.18632/oncotarget.2420 (PMC4279390; doi:10.18632/oncotarget.2420)
Supplement: Supplementary file 1 [file oncotarget-05-10518-s001.pdf]

SUPPLEMENTARY FIGURE AND TABLES

```
                   Signal Peptide
VEGFA206     1 MNFLLSWVHWSLALLLYLHHAKWSQAAPMAEGGGQNHHEVVKFMDVYQRSYCHPIETLVD
VEGFA172     1 MNFLLSWVHWSLALLLYLHHAKWSQAAPMAEGGGQNHHEVVKFMDVYQRSYCHPIETLVD

VEGFA206    61 IFQEYPDEIEYIFKPSCVPLMRCGGCCNDEGLECVPTEESNITMQIMRIKPHQGQHIGEM
VEGFA172    61 IFQEYPDEIEYIFKPSCVPLMRCGGCCNDEGLECVPTEESNITMQIMRIKPHQGQHIGEM

VEGFA206   121 SFLQHNKCECRPKKDRARQEKKSVRGKGKGQKRKRKKSRYKSWSVYVGARCCLMPWSLPG
VEGFA172   121 SFLQHNKCECRPKKDRARQEKKSVRGKGKGQKRKRKKSRYKSWSV---------------

VEGFA206   181 PHPCGPCSERRKHLFVQDPQTCKCSCKNTDSRCKARQLELNERTCRCDKPRR
VEGFA172   166 --PCGPCSERRKHLFVQDPQTCKCSCKNTDSRCKM
```

Supplementary Figure S1: Protein alignment of $VEGFA_{206}$ and the predicted amino acid sequence of $VEGFA_{172}$. $VEGFA_{172}$ isoform is 34 aa shorter than $VEGFA_{206}$ and ends with a methionine residue instead of an alanine. The first 26 aa correspond to the N-terminal signal peptide typical of secreted proteins.

Supplementary Table S1. RNA-sequencing data summary

	MSC				MSC+TGFα			
**Replicates**	**1**	**2**	**3**	**4**	**1**	**2**	**3**	**4**
**Total Reads**	39×10^6	35×10^6	36.5×10^6	39×10^6	49×10^6	43×10^6	45×10^6	50×10^6
**Mapped reads**	31×10^6	28.3×10^6	29.8×10^6	30.8×10^6	37.4×10^6	33.5×10^6	35.5×10^6	38.2×10^6
**Coverage (%)**	79.49	80.96	81.66	79.11	76.32	78.02	78.97	76.51

Supplementary Table S2. 10,068 differentially expressed genes between untreated and TGFα-treated MSCs

Supplementary Table S3. 1640 highly differentially regulated genes
